# Efficacy of combined tympanostomy tube insertion and adenoidectomy in the treatment of otitis media with effusion in children

**DOI:** 10.12669/pjms.40.11.10713

**Published:** 2024-12

**Authors:** Yongcai Weng, Yong Wu, Chunhua Hao, Yanjun Chu, Xiong Qian

**Affiliations:** 1 Yongcai Weng Department of Otorhinolaryngology, Jiaxing University Affiliated TCM Hospital, Jiaxing, Zhejiang Province 314001, P.R. China; 2 Yong Wu Department of Otorhinolaryngology, Jiaxing University Affiliated TCM Hospital, Jiaxing, Zhejiang Province 314001, P.R. China; 3 Chunhua Hao Department of Otorhinolaryngology, Jiaxing University Affiliated TCM Hospital, Jiaxing, Zhejiang Province 314001, P.R. China; 4 Yanjun Chu Department of Otorhinolaryngology, Jiaxing University Affiliated TCM Hospital, Jiaxing, Zhejiang Province 314001, P.R. China; 5 Xiong Qian Department of Pediatrics, Jiaxing University Affiliated TCM Hospital, Jiaxing, Zhejiang Province 314001, P.R. China

**Keywords:** Tympanostomy tube insertion, Adenoidectomy, Otitis media with effusion, Children

## Abstract

**Objective:**

To assess the efficacy of combined tympanostomy tube insertion (TTI) and adenoidectomy (Ad) *versus* Ad alone in the treatment of otitis media with effusion (OME) in children.

**Methods:**

Clinical data of 145 children with OME who underwent surgical treatment in Jiaxing University Affiliated TCM Hospital from January 2022 to November 2023, were retrospectively analyzed. Patients were grouped based on whether or not Ad was performed with TTI: children who underwent Ad alone were grouped as Ad group (n=71), and children who underwent a combined TTI and Ad were grouped as TTI+Ad group (n=74). Clinical efficacy, clinical indicators (recovery of hearing, disappearance of tinnitus, eardrum healing, and disappearance time of middle ear effusion), serum levels of inflammatory factors, recurrence, and incidence of complications were compared between the two groups.

**Results:**

Combined TTI+Ad resulted in a significantly better therapeutic effect three months after the surgery compared Ad alone (*P*<0.05). Combined treatment was associated with significantly shorter time needed for hearing recovery, disappearance of tinnitus, eardrum healing, and disappearance of middle ear effusion (*P*<0.05). Combined procedure significantly lowered levels of inflammatory factors (*P*<0.05), and was associated with lower postoperative recurrence rate (*P*<0.05) compared to Ad alone. There was no significant difference in the incidence of complications between the two methods of surgical OME treatment (*P*>0.05).

**Conclusions:**

Compared to Ad alone, combined TTI+Ad treatment is equally safe and more effective in the treatment of OME in children. This surgical approach is associated with reduced local inflammatory reactions and lower recurrence rate.

## INTRODUCTION

Otitis media with effusion (OME) in children occurs as a result of the obstruction of the eustachian tube, and may lead to complications such as deafness and tympanic effusion.^1^ The onset of the disease is often accompanied by tinnitus and hearing loss that may have serious impact on the quality of life of the affected children.^2^ Current treatment methods for OME in children mainly rely on pharmaceuticals, or surgical intervention.^1^–^3^ However, studies show that the drug therapy alone is often not effective enough: some OME patients have severe adenoid hypertrophy, which compresses the tympanic canal and pharyngeal opening, leading to recurrent disease attacks.^3^,^4^ Surgical methods that are routinely used for treating OME include tympanostomy tube insertion (TTI) and adenoidectomy (Ad).^4^-^7^ TTI is efficient in draining middle ear cavity effusion, and improving negative pressure state of the middle ear,^6^ while Ad is effectively removes compression of the tympanic canal and pharyngeal opening. Moreover, Ad is often performed using low-temperature plasma knives, which also allow to reduce the volume of bleeding.^7^

Studies have shown the efficacy of the combined TTI+Ad treatment for OME in children, and most studies focused on postoperative recurrence rate, with relatively few studies on inflammatory factor levels.^8^,^9^ In addition, whether the combined TTI+Ad treatment can reduce the incidence of postoperative complications compared with TTI or Ad alone remains controversial. This retrospective study aimed to analyze the efficacy of combined TTI+Ad surgery in treating OME in children, with a focus on the impact of the combined treatment on the postoperative recurrence, inflammatory response, and complications.

## METHODS

Clinical data of 145 children with OME, who underwent surgical treatment in Jiaxing University Affiliated TCM Hospital from January 2022 to November 2023, were retrospectively selected for the analysis. Patients were grouped based on whether or not Ad was performed with TTI: children who underwent simple Ad were assigned into the Ad group (n=71), and children who underwent a combination of TTI and Ad comprised the TTI+Ad group (n=74).

### Ethical Approval:

The ethics committee of our hospital has approved this study (No. SL-2023-0108, Date: November 29^th^ 2023).

### Inclusion criteria:

- Meets the diagnostic criteria for OME in children.^1^
- The ultrasound test results show a B- or C-shaped curve, the eardrum is inverted during otoscopy examination, and the hearing test shows abnormalities.
- Auditory brainstem response (ABR) examination detects waves I, III, and V, but the threshold is increased, the latency of wave I is prolonged, and the interval between waves I-V is normal.
- Electronic nasal endoscopy examination and nasopharyngeal lateral X-ray examination confirmed the presence of adenoid hypertrophy [adenoid/nasopharyngeal cavity (A/N) ≥ 0.6%].
- The patient is aged 3-10 years old, and pharmacological treatment has been ineffective for more than three months.
- Consented to Ad or TTI+Ad treatment.
- The clinical data is complete.

### Exclusion criteria:

- OME caused by nasopharyngeal tumors, meningoencephalocele, head or middle ear trauma.
- Congenital cleft lip and palate, congenital ciliary dysfunction syndrome, congenital or acquired immunodeficiency.
- Comorbidities and allergic constitution.
- History of chronic suppurative otitis media, history of ototoxic drugs, family history of deafness, history of middle and inner ear deformities, history of noise exposure, and other ear diseases.
- History of mental illness, cognitive impairment, severe liver and kidney dysfunction, etc.

### Surgical procedures:

### Adenoidectomy:

Tracheal intubation and intravenous combined general anesthesia were initiated. The child was placed in a supine position, with a thin pillow under the shoulders to tilt the head back. Oropharynx was exposed using a Davis mouthpiece. The 70° nasal endoscopic monitoring system was used to fully expose glandular body, Eustachian tube opening, round occipital, posterior nostril, and the top and side walls of the nasopharynx through the mouth. An electric cutter and synchronous suction were used to aspirate and remove adenoid tissue. During the operation, suction and cutting drill opening were always facing the gland, gradually progressing from top to bottom. Care was taken to remove glandular tissue around the occipital and pharyngeal opening of the Eustachian tube as thoroughly as possible, without damaging normal tissues to avoid postoperative scar narrowing of the pharyngeal opening of the Eustachian tube. Cotton ball compression was applied to the wound to stop the bleeding. In cases of active bleeding, plasma was used to stop the hemorrhage.

### Combined TTI and adenoidectomy:

After removal of adenoids, patient was placed with the affected ear in an upward position. Condition of tympanic effusion was assessed with the assistance of 0° ear endoscope. The incision of approximately 2.0 mm radially in the anterior or posterior inferior quadrant of the affected eardrum was done. A negative pressure suction device was used to completely drain the fluid in the drum chamber, a T-shaped tube was placed at the incision site and affixed at the edge of the incision. Instruments, such as ear endoscope, were then removed. Patient baseline data and relevant indicators were collected after three months of treatment and included:

### Data collection:

***(1) Clinical efficacy:*** It was divided into the following three levels.
***Full recovery:*** patient’s clinical symptoms (tinnitus, ear stuffiness) disappear, hearing returns to normal (hearing threshold less than or equal to 25dB), tympanic membrane markers return to normal, acoustic impedance tympanogram returns to type A, and stapedius reflex is normal.
***Effective:*** improved clinical symptoms, hearing threshold decreased by 10-15dB, tympanic membrane not significantly inverted, the acoustic impedance tympanogram changed from B-type to C-type/ transitioned from C-type to A-type/evidence of the increase in tympanic peak pressure on the C-type curve compared to preoperative state; increased acoustic compliance value of the A curve; stapedius reflex can be elicited and the reflex threshold decreases but does not reach normal values.
***Invalid:*** No significant improvement in clinical symptoms, no increase in hearing (no significant decrease in hearing threshold or below 10dB), and obvious retraction of the eardrum; obvious fluid accumulation in the middle ear cavity due to bulging of the eardrum, no change in the acoustic impedance tympanogram, and the stapedius reflex cannot be elicited. Total efficacy of the treatment was calculated as follows: Total effective rate = (recovery+effective) × total cases/100%.^10^
(2) Clinical indicators, including recovery of hearing, disappearance of tinnitus, eardrum healing, and disappearance time of middle ear effusion.
(3) Serum levels of inflammatory factors, including interleukin-6 (IL-6), IL-8 and tumor necrosis factor-alpha (TNF-α) were measured in 5mL of venous blood using fully automatic biochemical analyzer BK-280.
(4) Complications, including hearing loss, middle ear infection, tympanic sclerosis, and non-healing of tympanic membrane perforation.
(5) Recurrence, defined as decreased hearing and a sense of ear occlusion in patient that present with sunken and yellow, amber-like tympanic membrane, with an intratympanic pressure of < -200 mm H_2_O.

### Statistical analysis:

SPSS version 25.0 (IBM Corp, Armonk, NY, USA) was used for analysis. For continuous variables, mean and standard deviation (SD) were calculated. For categorical variables, frequency distribution was provided and expressed as a percentage. The chi square test was used to compare categorical variables between the two groups. Independent sample *t*-test was used to compare the means of two independent samples, especially for continuous variables. The hypothesis of equal variance was examined and considered in the analysis. A *p*-value less than 0.05 was considered an indicator of statistically significant differences.

## RESULTS

A total of 145 patients (76 males and 69 females) were retrospectively included in the study. Age of participants ranged from three to 10 years, with a mean of 5.47 ± 1.58 years. There were 71 patients in the Ad group, and 74 patients in the TTI+Ad group, with no significant difference in baseline data between the two groups (*P*>0.05.) Table-I. After the treatment, total efficacy of the TTI+Ad group was significantly higher than that of the Ad group (*P*<0.05) Table-II. Combined TTI/Ad surgery was associated with significantly shorter times of postoperative hearing recovery, tinnitus disappearance, eardrum healing, and middle ear effusion disappearance compared to Ad alone (*P*<0 05) Table-III. Before the treatment, there was no significant difference in the serum levels of inflammatory factors between the two groups. After the treatment, levels of IL-6, IL-8 and TNF-α levels in the TTI+Ad group were significantly lower than those in the Ad group (*P*<0 05) Table-IV. TTI+Ad surgery was associated with significantly lower recurrence rate compared to Ad alone (*P*<0 05), while there was no significant difference in the incidence of complications between the two methods (*P*>0.05). Table-V

**Table-I:** Comparison of baseline data between two groups of patients.

Baseline data	Ad group (n=71)	TTI+Ad group (n=74)	χ ^2^/t	P
Male/female	35/36	41/33	0.542	0.461
Age (year)	5.58±1.64	5.36±1.51	0.811	0.419
Disease duration (months)	8.58±2.63	8.80±2.18	-0.549	0.584
Affected ear			1.398	0.237
Monaural	23(32.39)	31(41.89)		
Binaural	48(67.61)	43(58.11)		
ABR threshold (dB)	55.87±6.51	54.78±6.09	1.054	0.294
A/N			0.137	0.711
0.71~0.61	29(40.85)	28(37.84)		
>0.71	42(59.15)	46(62.16)		

**Table-II:** Comparison of clinical efficacy between two groups.

Group	n	Recovery	Effective	Invalid	Total effective rate (%)
Ad group	71	20 (28.17)	36 (50.70)	15 (21.13)	78.87
TTI+Ad group	74	29 (39.19)	41 (55.41)	4 (5.41)	94.59
*χ^2^*					9.413
*P*					0.001

**Table-III:** Comparison of clinical indicators between two groups (day).

Group	Recovery of hearing	Disappearance of tinnitus	Eardrum healing	Disappearance of middle ear effusion
Ad group (n=71)	27.90±4.82	12.83±2.66	16.96±2.91	11.92±2.61
TTI+Ad group (n=74)	25.69±5.40	9.59±1.71	12.49±1.87	7.61±1.82
*t*	2.598	8.669	11.949	11.479
*P*	0.01	<0.001	0.321	<0.001

**Table-IV:** Comparison of serum inflammatory factor levels before and after treatment between two groups.

Group	IL-6 (ng/ml)		IL-8 (ng/ml)		TNF-α(μg/L)	

	Before treatment	After treatment	Before treatment	After treatment	Before treatment	After treatment
Ad group (n=71)	33.76±5.09	21.66±2.83	536.18±60.87	334.96±45.58	0.80±0.14	0.48±0.11
TTI+Ad group (n=74)	34.77±4.72	19.74±2.87	526.50±56.24	277.22±39.16	0.79±0.13	0.26±0.09
*t*	-1.239	4.048	0.995	8.193	0.536	12.913
*P*	0.217	<0.001	0.321	<0.001	0.593	<0.001

**Table-V:** Comparison of postoperative complication rates between two groups.

Group	Recurrence rate (%)	Complications (%)				

		Hearing loss	Middle ear infection	Tympanosclerosis	Non-healing of tympanic membrane perforation	Total
Ad group (n=71)	10 (14.08)	2 (2.82)	1 (1.41)	2 (2.82)	1 (1.41)	6 (8.45)
TTI+Ad group (n=74)	3 (4.05)	1 (1.35)	1 (1.35)	0 (0)	1 (1.35)	3 (4.05)
*χ^2^*	*4.467*					0.566^a^
*P*	*0.035*					0.452

a , Yates’s correction for continuity.

## DISCUSSION

The results of this study indicate that combined TT/Ad surgical treatment of OME in children is significantly more effective than Ad alone. Our results demonstrated that TTI+Ad was associated with lower levels of serum inflammatory factors, and low recurrence rate, with no significant increase in complications. OME is a frequent and common otolaryngological disease that is characterized by the presence of non-purulent lesion of the middle ear.^1^,^2^,^11^ Current guidelines indicate that in OME patients with adenoid hypertrophy, surgical intervention is necessary when conservative treatment is ineffective. Recently, combination of Ad and TTI has become a commonly used method for the treatment of OME accompanied by adenoid hypertrophy, since such patients require both adenoidectomy, and restoring middle ear ventilation to improve the function of the Eustachian tube.^8^,^12^-^14^ Nie Lei et al.^13^ reported that TTI+Ad has a significant therapeutic effect in patients with OME, and is associated with low levels of inflammatory factors, fewer complications, and lower recurrence rate.

A meta-analysis by Mikals SJ et al.^14^ showed that Ad, as an auxiliary method for first-time TTI, can reduce the risk of repeat surgery in children over four years old. A study by Shareef et al.^8^ that included 409 patients showed that TTI+Ad method can further improve middle ear function, reduce the risk of repeated surgery and complications in young (under four years old) patients with OME. In agreement with these results, our study that included children aged 3-10 years old, further confirmed the value of the combined TTI+Ad surgery in this group of patients.^8^,^9^

Previous studies have suggested that the mechanisms by which adenoid hypertrophy affects the cure rate of OME include middle ear effusion, increased levels of mast cells, inflammatory mediators, and local inflammatory reactions.^15^,^16^ TTI can effectively drain the fluid in the tympanic cavity by incising the periosteum, achieving pressure balance inside and outside the Eustachian tube. Additionally, placing a T-shaped tube at the tympanic membrane can prevent the tympanic cavity from accumulating fluid again, thus improving hearing and alleviating symptoms.^10^,^16^ Children with OME often present with large amounts of fluid accumulation.^2^ Adenoid hypertrophy that is accompanied by the increase in inflammatory factors, leads to further decline in the function of the Eustachian tube and subsequent fluid accumulation.^1^,^2^,^12^

Indeed, Wang et al.^17^ showed that TTI perforation can effectively remove inflammatory factors in middle ear effusion and inhibit the release of inflammatory factors. Furthermore, Ad can reduce the volume of adenoid hypertrophy, lower the bacterial load, reduce proliferation of T and B lymphocytes, and reduce level of inflammatory factors.^15^,^16^,^18^ As shown by Nguyen LH et al.,^19^ TTI+Ad can effectively reduce IL-6 and IL-8 levels in patients, while Ad significantly inhibits generation of inflammatory factors, alleviating the inflammatory response of children, and accelerating the recovery. Our results demonstrate that serum levels of IL-6, IL-8 and TNF-α in the TTI+Ad group were significantly lower than those in the Ad group, which is consistent with the previous research.

Our study further confirms that TTI+Ad treatment is safe and can effectively reduce levels of inflammatory mediators in patients, alleviate their inflammatory response, and have a good effect on postoperative recovery of OME in children,^19^ which is similar to the results of Ren L.^20^ Combined surgical treatment, therefore, not only significantly alleviates excessive secretion and effusion in the ear cavity, but also improves the symptoms of adenoid hypertrophy, which is highly beneficial for improving the hearing of patients.^19^,^20^ The results of our study also indicate that the postoperative recurrence rate after the combined surgery was 4.05%, significantly lower than 14.08% in patients who underwent Ad alone. There was no significant increase in complications in the TTI+Ad group. We may speculate that this effect is due to the ability of TTI to effectively clear the secretion of the patient’s tympanic cavity, quickly relieve the negative pressure of the tympanic cavity, improve the function of the patient’s Eustachian tube, and thus improve the patient’s clinical symptoms and characteristics.^18^,^21^ At the same time, Ad completely eliminates locally persistent infection foci, relieves mechanical obstruction, ensures unobstructed drainage of the Eustachian tube and middle ear cavity, and effectively prevents retrograde flow of the Eustachian tube.^5^,^19^-^21^ Together, these procedures significantly improve postoperative quality of life of the patient, accelerate recovery, and reduce inflammatory reactions and recurrence. Therefore, in clinical practice, under the premise that both Ad and TTI can be used, the combination of the two should be given priority for children because the combined treatment has a more ideal therapeutic effect.

### Limitation:

Firstly, this is a single center retrospective analysis with a small sample size and selection bias. Secondly, only clinical effects after three months of surgery were analyzed, and a longer follow-up time is needed to verify the effectiveness of TTI+Ad. Further prospective studies, as well as studies that specifically focus on children under the age of four are needed.

## CONCLUSION

Compared to adenoidectomy alone, TTI+Ad surgery has a more profound treatment effect on OME in children, and is associated with lower serum levels of inflammatory factors, lower recurrence rate with no significant increase in complications.

### Authors’ contributions:

**YW:** Conceived and designed the study, prepared the manuscript.

**YWU**, **CH**, **YC** and **XQ:** Collected the data, performed the analysis and critical Review.

All authors have read and approved the final manuscript and are responsible for the integrity of the study.

Zhejiang Province Traditional Chinese Medicine Science and Technology Plan Project (2024ZL1050).
